# Post-COVID syndrome: A prospective study in a tertiary hospital of Nepal

**DOI:** 10.1371/journal.pone.0272636

**Published:** 2022-08-10

**Authors:** Sangam Shah, Shreeyash Raj Bhattarai, Kriti Basnet, Yagya Raj Adhikari, Tara Ballav Adhikari, Nikita Bhatta, Rajan Chamlagain, Susan Aryal, Sanjit Kumar Sah, Govinda Bhandari, Bibek Bhandari, Sujan Poudel, Pankaj Pant, Santa Kumar Das

**Affiliations:** 1 Maharajgunj Medical Campus, Institute of Medicine, Tribhuvan University, Maharajgunj, Kathmandu, Nepal; 2 Tribhuvan University Teaching Hospital, Maharajgunj, Kathmandu, Nepal; 3 Department of Public Health, Aarhus University, Aarhus, Denmark; 4 Nepal Health Frontiers, Kathmandu, Nepal; 5 COBIN Project, Nepal Development Society, Chitwan, Nepal; 6 Maharajgunj Nursing Campus, Institute of Medicine, Tribhuvan University, Kathmandu, Nepal; 7 Department of Internal Medicine, Institute of Medicine, Tribhuvan University, Kathmandu, Nepal; 8 Division of Research and Academic Affairs, Larkins Community Hospital, South Miami, Florida, United States of America; 9 Department of Pulmonology and Critical Care, Maharajgunj Medical Campus, Institute of Medicine, Tribhuvan University, Kathmandu, Nepal; Kasturba Medical College Manipal, INDIA

## Abstract

**Introduction:**

The post-coronavirus disease 2019 (COVID-19) syndrome is defined as the persistence of symptoms after viral clearance and the emergence of new symptoms after a few months following recovery from COVID-19. This study aimed to assess the prevalence of post-COVID-19 syndrome and the risk factors that contribute to its development.

**Methods:**

This study was conducted prospectively in Tribhuvan University Teaching Hospital (TUTH), located in Maharajgunj, Kathmandu. The patients were followed up for three months.

**Results:**

The post-COVID status of 300 patients admitted to the COVID emergency of TUTH was studied. The mean age of the patients was 46.6±15.7 years, and the proportion of male (56%) was slightly higher than female (44%). Most of the patients (81.7%) had fever on their presentation to the emergency which was followed by fatigue (81.3%) and cough (78.3%). During the post-COVID phase, fatigue was the most common persistent symptom, with 34% experiencing fatigue after 60 days and 28.3% even after 90 days from the onset of symptoms. Univariate logistic regression showed sore throat (OR 4.6; 95% CI (2.8–7.6)), rhinitis (OR 3.6; 95% CI (2.1–5.9)), fatigue (OR 3.7; 95% CI (1.8–7.6)), diarrhea (OR 4.1; 95% CI (2.4–6.9)), anosmia (OR 6.7; 95% CI (3.9–11.3)), ageusia (OR 7.8; 95% CI (4.5–13.4)) and shortness of breath (OR 14.9; 95% CI (1.8–119.6)) at admission were all predictors of post-COVID syndrome after three months.

**Conclusion:**

Even after recovering from COVID-19, people with COVID-19 may develop symptoms. As a result, COVID-19’s long-term consequences should not be neglected, as they may lead to increased morbidity among patients, consumption of financial resources, and added burden on the health system.

## Introduction

Millions of people worldwide have been affected by the COVID-19 pandemic, which has wreaked havoc on their health and economic well-being. Although many people died from the sickness, patients with milder symptoms were more common [1]. Those who acquired mild to moderate symptoms were reported to be entirely recovered after about a week [2, 3]. However, there has been a persistence or emergence of new symptoms following the recovery from COVID, even among those who initially had mild disease. This is termed as a post-COVID syndrome [3, 4]. According to the study by Greenhalgh et al. in the UK, about 10% of patients, were sick for three weeks, and a small proportion of the population persisted for months [5].

Post-COVID syndrome is a multisystem disorder that develops after an acute episode of illness. Many studies of past respiratory virus pandemics show the persistence of diverse symptoms after an acute episode, which means post-COVID syndrome is not a new entity [6, 7]. There is no clear evidence suggesting the exact pathogenesis for the development of chronic post-COVID illness. One study shows the role of mast cell activation in developing long-term symptoms [8]. The symptoms may be mild such as fatigue, cough, alopecia, shortness of breath, or severe, leading to stroke, renal failure, cardiac abnormalities, etc. [4]. Its prevalence was more common among those who developed severe diseases requiring hospital admission [9, 10].

The complete clinical picture of the post-COVID syndrome is complicated and poorly understood. However, because of the viral tropism defined by viral entrance into cells via a broadly expressed ACE2 receptor, numerous organs may be susceptible to both acute and chronic harm, further complicating the already complex clinical picture of post-COVID syndrome.

The post-COVID syndrome is of concern for public health in the face of an existing pandemic. However, there is a lack of evidence on its pathogenesis and management. We need an evidence-guided holistic approach for the overall management of this entity. This study focuses on collecting information about the patterns of symptoms in long-term COVID patients and their association with the severity of the disease.

## Methods

### Study design and setting

This study was conducted prospectively in Tribhuvan University Teaching Hospital (TUTH), located in Maharajgunj, Kathmandu. This center was chosen for study because of its high flow of patients during the second wave of the COVID-19 pandemic in Nepal. Ethical approval for conducting the study was taken from the Institutional Review Board (IRB) of TUTH, Institute of Medicine (IOM). [Approval number: 65 (6–11) E^2^ 078/079]

### Study participants and eligibility criteria

**Fig 1:**
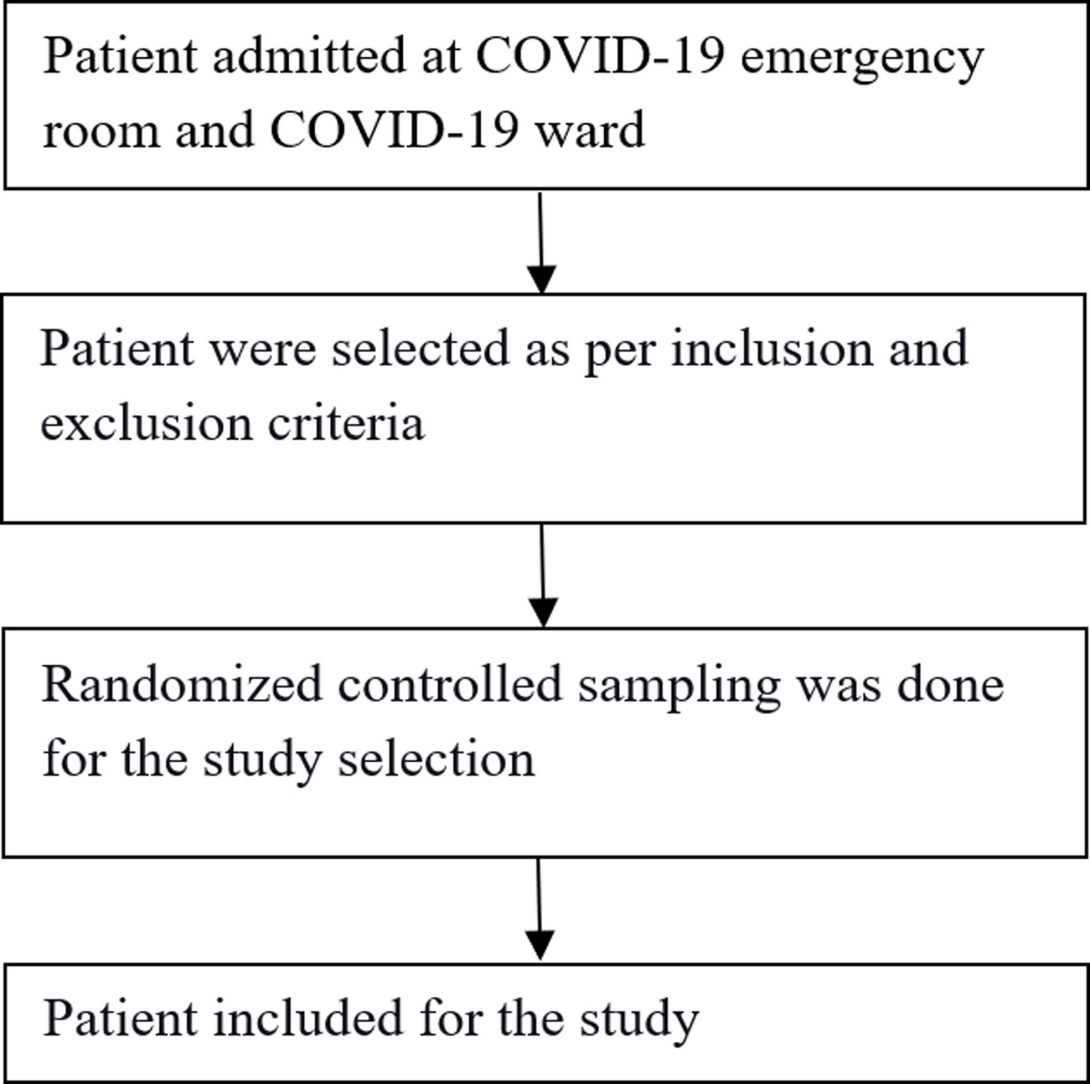
Study flowchart for selection of study participants.

The patients were selected randomly from the ones who visited the COVID-19 emergency room and COVID-19 ward of TUTH. Patients admitted to the emergency room from May 2021 to July 2021 were selected for the study due to the peak flow of patients during the second wave of the COVID-19 pandemic in Nepal. The admitted patients and those discharged from the emergency room were followed up for three consecutive months from the admission date Fig 1.

#### Inclusion criteria

Only those patients of age ≥18 years who were diagnosed SARS-CoV2 positive by reverse transcriptase polymerase chain reaction (RT-PCR) were selected for the study. Patients were selected irrespective of their symptoms seen in COVID-19.

#### Exclusion criteria

All patients below 18 years of age and not of Nepalese nationality were excluded from the study. We also excluded all patients who showed positive in other tests rather than on the RT-PCR test. Patients who did not survive after admission to the hospital were also excluded from the study.

### Sampling

We performed randomized sampling among the patients admitted to the COVID-19 emergency and COVID-19 ward. The patients’ list was taken from the COVID-19 emergency and ward record book, and participants were chosen using simple random sampling. Using Excel’s rand command, random values were generated for each patient, and those with the highest values were selected to participate.

### Study tools and techniques

A structured questionnaire was used to collect the data prospectively from the admitted patients. Written informed consent was taken from all the patients. The patients who did not follow up in the consecutive months were contacted through phone calls at the end of successive months from the day of their admission.

### Study variables

The variables were categorized under socio-demographic factors, personal factors, and other study variables. The age and sex of the patient were included under sociodemographic factors. Similarly, patients’ comorbidities, smoking, and alcohol consumption habits were recorded under personal characteristics. Both daily and occasional users were considered under the heading. Other study variables include symptoms present during the SARS-CoV2 positive phase, duration of symptoms, length of hospital stay, ward admitted during the stay, vaccination status of the patient, the severity of infection, persistent positivity after 14 days of infection, and symptoms present at the end of first, second and third month after admission of the patient. Post-COVID syndrome is described as a set of persistent clinical signs and symptoms that emerge during or after exposure to COVID-19, last more than 12 weeks, and are not explained by any other diagnosis.

### Statistical analysis

Data was compiled, edited, and checked daily to maintain consistency. The data was collected in Microsoft Excel (Ver. 2013). For statistical analysis, SPSS 21 (IBM Corp. Released 2012. IBM SPSS Statistics for Windows, Version 21.0. Armonk, NY: IBM Corp.) was used. Descriptive analysis was done to identify the distribution of socio-demographic characteristics of patients, and the association was measured using a parametric and non-parametric test (depending upon the distribution of data). Univariate and multivariate logistic regression were used to identify predictors of the post-COVID syndrome. A p-value <0.05 for the two-tailed test was considered statistically significant.

## Results

### Study population and sociodemographic profile

We studied the post-COVID status of 300 patients admitted to the COVID emergency of TUTH, IOM, for three consecutive months since the day of PCR positivity of the patients. The mean age of the patients was 46.6±15.7, comprised of patients as low from 18 years of age to high up to 88 years of age. The proportion of males (56%) was slightly higher than females (44%), with the majority of them were unemployed/dependent(37%). The average length of hospital stay for patients was 8.1±5.9 days. Out of the 300 patients, 224 (74.7%) were admitted to the general ward, 26 (8.7%) in the high dependency unit ward, and 11 (3.7%) in the intensive care unit. Only 39 (13%) of the patients were sent for home isolation as most of the patients fell under the moderate (42.7%) and severe (23.7%) category were admitted.

Among total patients, 62.7% had one of the seven comorbidities studied, including 28.7% with hypertension and 18.3% with diabetes mellitus. 22.3% of our study population were long-term smokers, and 36.7% were alcohol consumers for >10 years. The demographic details of the included patient are shown in Table 1.

**Table 1:** Socio-demographic profile of included patients.

	Mean	SD
Age in years	46.6 (18–88)	15.7
Duration of hospitalization (in days)	8.1 (4–45)	5.9
	**Frequency**	**Percent**
**Sex**		
Male	168	56.0
Female	132	44.0
**Occupation**		
Business man	53	17.7
Service	92	30.7
Labor	21	7.0
Dependent	111	37.0
Others	23	7.7
**Smoker**	67	22.3
**Alcohol**	110	36.7
**Hospital stay**		
General ward	224	74.7
HDU	26	8.7
ICU	11	3.7
Home isolation	39	13.0
**No of people received Vaccine**	76	25.3
**Name of vaccine**		
COVISHIELD	32	10.7
Vero Cell	15	5.0
**Number of doses**		
First dose	16	5.3
Second dose	31	10.3
**Comorbidities**		
Hypertension	86	28.7
DM	55	18.3
COPD	14	4.7
Pulmonary TB	4	1.3
Malignancy	2	0.7
Autoimmune	2	0.7
Thyroid disorder	25	8.3

### Patient symptoms during acute and post-COVID phase

Most of the patients (81.7%) had fever on their presentation to the emergency room, which was followed by fatigue (81.3%) and cough (78.3%). Few patients also presented with diarrhea (6.7%), rhinitis (6.7%), and headache (3.3%). 56.7% of patients re-did their PCR test 14 days after the initial test, and 24.7% of them tested positive again. 26% of the patients required no treatment in the hospital and were only under observation. 6.7% of the patients were treated with Remdesivir, and 12 patients (4%) were treated with tocilizumab. Most patients with moderate to severe severity were treated with steroids (63.3%).

During the post-COVID phase, fatigue was the most common persistent symptom, with 34% experiencing fatigue after 60 days and 28.3% even after 90 days from the onset of symptoms. Also, 6.7% continued having some shortness of breath after three months of symptoms onset. The new onset of alopecia in the following months of acute infection was found in 18.3% of the patients. Other uncommon post-COVID symptoms were diarrhea (0.3%), sore throat (0.3%), cough (2%), anosmia (4.3%) and ageusia (4.3%). We also found that the prevalence of post-COVID syndrome was higher in moderate and severe cases as 29.7% of all the mild cases, 42.2% of all the moderate cases and 47.7% of all the severe cases developed the post-COVID syndrome. The symptoms in the acute phase and the following consecutive months are given in Table 2.

**Table 2:** Symptoms of patient at each visit.

Parameter	Acute phase		First visit		Second visit		Third visit	
	n	%	n	%	n	%	n	%
**Fever**	261	81.7	14	4.7	0	0	0	0
**Cough**	235	78.3	91	30.3	28	9.3	6	2.0
**Sore throat**	137	45.7	37	12.3	10	3.3	1	0.3
**Rhinitis**	87	29.0	20	6.7	3	1.0	0	0
**Fatigue**	244	81.3	171	57.0	102	34.0	85	28.3
**Anosmia**	152	50.7	92	30.7	43	14.3	13	4.3
**Ageusia**	155	51.7	87	29.0	41	13.7	13	4.3
**Headache**	92	30.7	10	3.3	2	0.7	0	0
**Diarrhea**	85	28.3	20	6.7	2	0.7	1	0.3
**Alopecia**					47	15.7	55	18.3
**Shortness of breath**	198	66.0	98	22.3	35	11.7	20	6.7
**Persistent positivity**								
Yes	42	14.0	5	1.7	0	0	0	0
No	128	42.7	91	30.3	82	27.3	83	27.7
No PCR done	130	43.3	204	68.3	218	72.7	217	72.3
**Severity**								
Mild	101	33.7	-	-	-	-	-	-
Moderate	128	42.7	-	-	-	-	-	-
Severe	71	23.7	-	-	-	-	-	-
**Treatment**								
No treatment	78	26.0	270	90.0	296	98.7	300	100
Remdesivir	20	6.7	0	0	0	0	-	-
Tocilizumab	8	2.7	0	0	0	0	-	-
Steroids	194	64.7	30	10.0	4	1.3	-	-
**Interval of symptom for first visit**			**Second visit**		**Third visit**		**-**	**-**
from beginning	210	70.0	157	52.3	101	33.7	-	-
<7 days	38	12.7	13	4.3	18	6.0	-	-
> 7 days	52	17.3	130	43.3	181	60.3	-	-

n is frequency

We found that fatigue and alopecia was present as a post-COVID syndrome irrespective of the initial severity of disease in the acute phase as 21.8% of the mild, 28.1% of the moderate, and 38% of the severe cases had fatigue, and 12.9% of calm, 19.5% of the average and 23.9% of the extreme cases had alopecia after three months. None of the mild cases developed a cough as a post-COVID syndrome. However, 3.1% of the moderate cases and 2.8% of the severe cases still had coughs even at the end of the third month. The sore throat was also present in one person after three months, who had severe symptoms in the acute phase. No patient had fever, rhinitis, and headache at the end of the third month. The table showing the distribution of post-COVID syndrome about the severity of disease in the acute phase is given in Table 3.

**Table 3:** Severity of symptoms at third visit.

Symptoms	Severity		
	Mild	Moderate	Severe
**Fever**	0	0	0
**Cough**	0	4 (3.1%)	2 (2.8%)
**Sore throat**	0	0	1 (1.4%)
**Rhinitis**	0	0	0
**Fatigue**	22 (21.8%)	36 (28.1%)	27 (38%)
**Anosmia**	5 (5%)	5 (3.9%)	3 (4.2%)
**Ageusia**	6 (5.9%)	5 (3.9%)	2 (2.8%)
**Headache**	0	0	0
**Diarrhea**	0	0	1 (1.4%)
**Alopecia**	13 (12.9%)	25 (19.5%)	17 (23.9%)
**Shortness of breath**	2 (2%)	10 (7.8%)	8 (11.3%)

### Univariate and multivariate analysis

A total of 118 (39.3%) patients with, 68 male and 50 female, had post COVID-19 symptoms at the end of the third month from the day of the PCR positive test. Univariate logistic regression showed sore throat (OR 4.61; 95% CI (2.8–7.6)), rhinitis (OR 3.55; 95% CI (2.1–5.9)), fatigue (OR 3.65; 95% CI (1.76–7.56)), diarrhea (OR 4.11; 95% CI (2.42–6.9)), anosmia (OR 6.68; 95% CI (3.94–11.34)), ageusia (OR 7.77; 95% CI (4.51–13.38)) and shortness of breath (OR 14.94; 95% CI (1.8–119.5)) at admission were all predictors of post-COVID syndrome after three months. In multivariate logistic regression, the development of symptoms like fatigue (OR 0.38; 95%CI (0.15–0.97)), headache (OR 2.17; 95%CI (1.09–4.29)), ageusia (OR 0.27; 95%CI (0.10–0.71)) and shortness of breath (OR 0.53; 95%CI (0.29–0.97)) at admission were found to be independent predictors for developing post-COVID syndrome after three months. The details of the predictor of the post-COVID syndrome are shown in S1 Table.

## Discussion

The majority of SARS-CoV-2-convalescent patients in this large prospective trial of 300 individuals initially presented with mild symptoms (WHO clinical progression scale score 1–3) [11]. After four months, we discovered that 39.3% (118/300) of the participants had long-term health issues after the initial recovery from the disease, the symptoms developed in several ways. The symptoms were diverse, and some individuals had symptoms from both COVID-19 and post-COVID-19 syndrome. To our knowledge, this is the first set of data in which a large group of SARS-CoV-2-convalescent patients was prospectively followed up using a standardized procedural questionnaire for a median of three months.

Recently, it has been clear that certain patients, regardless of disease severity, continue to have symptoms weeks and months after the onset of COVID-19 [12–14]. There is no agreement on whether symptoms remain or arise again in the post-COVID-19 state, and the condition is not defined. Greenhalgh et al. [5]. attempted to distinguish between “post-acute COVID-19” and “chronic COVID-19,” which can last longer than three weeks and 12 weeks, respectively, after the onset of the first symptoms. We chose the term post-COVID syndrome instead of post-acute COVID-19 syndrome because the symptoms lasted longer than three months. Furthermore, no precise clinical case definition of the post-COVID syndrome has been established [15]. The majority of the data came from hospitalized patients with serious illnesses [16–18]. The time span used to identify the syndrome in recent studies ranged from >28–30 days [12, 19], 60 days [12, 17, 19], to more than three months [12, 17]. However, rather than a critical examination of actual symptoms, time durations were determined on the maximum follow-up time of the individual cohorts. Previously, no systematic evaluation of which symptoms characterize post-COVID syndrome had been conducted. Shortness of breath [17], fatigue, joint pain [17], anosmia [17, 20, 21], and ageusia [14] are the most typically reported and hence linked to post-COVID syndrome. Two-thirds of hospitalized patients had a lower quality of life due to these symptoms [14, 17].

The demographics of patients with COVID-19 in this study varied from those from Western countries. The patient’s age was the most noticeable feature. In this study, 21.3% of patients were aged 60 years. In a study from the USA, 31% of patients were aged >65 years [22]. This is probably due to the socio-cultural background of Nepal, where the proportion of the elderly population (8.1%) is lower than that in the Western world (North America, 16%; Europe, 21%) [23]. The proportion of men with COVID-19 was higher than that of women in this study. Most patients initially appeared with fever, cough, anosmia, hypoxia, and lethargy. Previous research has found a similar tendency [24, 25]. The duration of illness was six days on average, which is similar to what was found in a prior study [26]. Hypertension, diabetes mellitus-II, chronic lung disease, and malignancies were the most common concomitant conditions before SARS-CoV-2 infection. They thus reflected the most common concomitant diseases in the general population [27, 28] as well as those reported in other studies with comparable SARS-CoV-2-convalescent cohorts [29, 30].

The most prevalent symptom was post-viral fatigue (28.3%), followed by alopecia, exertional dyspnea, ageusia, and anosmia. Fatigue has been identified as one of the most common symptoms of post-COVID syndrome [20, 31] and among SARS-CoV-1 pandemic survivors in 2003 [32]. Endothelial failure in brain capillaries, recently revealed by Nauen et al., [33] could represent a physical correlate for fatigue. We found that even individuals with initially moderate symptoms can acquire fatigue as a prominent symptom of post-COVID syndrome [31], which is consistent with Townsend et al. findings. It should be noted, however, that fatigue varies from person to person, and no single test can prove a fatigue diagnosis.

Similarly, anosmia is a well-known symptom in COVID-19 patients [30, 34, 35] which appears to be more common in women [29, 36] and improves with time [29]. Anosmia’s pathophysiological mechanisms aren’t entirely known. Injury to the olfactory neuroepithelium is possible because SARS-CoV-2 accesses the nasal epithelium via the Angiotensin-converting enzyme-2 (ACE-2) receptor [37]. Female sex, respiratory distress, a lengthy recovery period, and the severity of the disease were all identified as risk factors for the post-COVID-19 syndrome. As a result, even after apparent clinical recovery, the patients did not fully heal, according to this study. Moreover, a third of the patients experienced long-term consequences and distress from COVID-19 infection.

Among the patients hospitalized in this study, 39.3% had the post-COVID syndrome. To meet the WHO recovery guidelines, these individuals needed a long period to recover. Carfi et al. [17] conducted a follow-up research on patients who met the WHO criteria for avoiding quarantine (no fever for three days in a row, improvement in other symptoms, and two negative SARS-CoV-2 test results 24 hours apart). They discovered that at least one symptom persisted in about 87%. The study sample was modest, however, and a significant number of patients required intensive care. As a result, the research stated above are insufficient to explain the post-COVID scenario. Furthermore, a large number of the patients in the study were admitted to the intensive care unit. Post-intensive care syndrome can cause symptoms like executive dysfunction, anxiety, sadness, and post-traumatic stress disorder in patients hospitalized to the intensive care unit [38].

As a result, we tried to characterize the disorder by adding individuals with mild, moderate, and severe disease who could develop post-intensive care syndrome. We found that almost half of the patients developed new symptoms, had mild COVID symptoms that persisted, or had chronic conditions that had worsened. Even after three weeks of COVID-19 positive, 35% of patients did not return to their previous health state, according to a study from the United States [39]. In another study, over 90% of COVID-19-positive hospitalized patients had at least one symptom persisting after two months of discharge; 12.6% had no relevant symptoms, 32% had one or two symptoms, and 55% had three or more symptoms [17]. In our study, 43.7% of the patients reported at least one persistent symptom, and 56.30% had multiple persistent symptoms.

Various studies have identified a variety of post-COVID-19 symptoms. Even in moderate situations, post-COVID19 symptoms might occur [17]. The most common symptoms described in most studies were fatigue, cough, respiratory discomfort, and headache [5, 17, 40]. Fatigue, persistent cough, exertional dyspnea, and body discomfort or vertigo were seen in 28.3%, 2%, 6.7%, and 17.7% of cases, respectively, in our study. The cause of fatigue’s predominance was mostly unknown. Fatigue may be caused by immune system changes caused by viral infections [6, 39]. Persistent squeal lung injury can explain cough and respiratory difficulty. Detected shortness of breath and anosmia in the context of post-COVID syndrome may be attributed to SARS-CoV-2 pathomechanism: vascular angiogenesis was observed at a higher frequency in the lungs of patients with COVID-19 when compared to individuals infected with influenza virus [41]. Gaebler et al. recently looked at intestinal samples taken from asymptomatic patients three months following the onset of COVID-19 [42]. SARS-CoV-2 was shown to be persistent in the small intestine of 7 out of 14 individuals [43]. Because the intestine is the largest lymphatic organ [44], residual virus particles in the intestine could be linked to long-term effects.

Post-COVID syndrome has a complex clinical picture that is still being explored. However, because of the viral tropism defined by viral entrance into cells through a widely expressed ACE2 receptor [37], numerous organs may be susceptible to both acute and chronic damage, further complicating the post-COVID syndrome clinical picture [45]. Effective or causative medicines have yet to be discovered.

## Strengths and limitations

In contrast to previous studies, ours has few advantages. Our sample comprises mild to moderate to severe COVID-19 individuals who have been monitored for a median of three months. Similarly, at each visit, all patients were examined by at least one trained physician to critically assess all reported symptoms and complement information from the written questionnaires with a detailed medical history and physical examination whenever possible. The alternate diagnosis like secondary bacterial or fungal pneumonia or fibrosis, or obstructive airway disease, which could explain the prolonged symptoms, was not taken into consideration. Despite this, our study has some limitations. A telephonic interview was used to follow up with patients. As a result, a proper evaluation of the patient’s quality of life was impossible. Furthermore, most associated factors had small effect sizes. To establish a strong correlation, bigger sample size is required. C-reactive protein (CRP), D-dimers, creatinine, and other common laboratory values were not evaluated.

## Conclusion

The prevalence of post-COVID-19 symptoms was seen in 39.3% (95% CI 33.8–44.8) of patients in a tertiary hospital of Nepal. As a result, COVID-19’s long-term consequences should not be neglected, as they may lead to increased morbidity among patients, consumption of financial resources, and increased health system burden.

## Supporting information

S1 Table Univariate and multivariate analysis of associated symptoms. (DOCX)
