# Supplementary material for: Post-COVID syndrome: A prospective study in a tertiary hospital of Nepal
Source: PLoS One. 2022 Aug 10;17(8):e0272636. doi: 10.1371/journal.pone.0272636 (PMC9365179; doi:10.1371/journal.pone.0272636)
Supplement: S1 Table — (DOCX) [file pone.0272636.s001.docx]

**Supplementary table 1: Univariate and multivariate analysis of associated symptoms**

|  | **Post-COVID syndrome** | | **Univariate regression** | | **Multi-Variable regression** | |
| --- | --- | --- | --- | --- | --- | --- |
|  | **No** | **Yes** | **OR (95% CI)** | **p-value** | **aOR (95%CI)** | **p-value** |
| Male | 100, 54.9% | 68, 57.6% | 0.897 (0.56-1.43) | 0.640 |  |  |
| Female | 82, 45.1% | 50, 42.4% |  |  |  |  |
| Preconditions | 65, 35.7% | 55, 46.6% | 1.57 (0.98-2.51) | 0.060 | 0.59 (0.33-1.06) | 0.08 |
| Symptoms | | | | | | |
| Fever | 160 87.9% | 101 85.6% | 0.82 (0.41-1.61) | 0.560 | 1.43 (0.61-3.39) | 0.409 |
| Cough | 137, 75.3% | 98, 83.1% | 1.60 (0.89-2.89) | 0.110 | 1.9 (0.8-4.4) | 0.098 |
| Sore throat | 57, 31.3% | 80 67.8% | 4.61 (2.8-7.6) | **<0.001** | 0.50 (0.23-1.08) | 0.080 |
| Rhinitis | 34, 18.7% | 53, 44.9% | 3.55 (2.1-5.9) | **<0.001** | 0.56 (0.28-1.11) | 0.100 |
| Fatigue | 136, 74.7% | 108, 95.1% | 3.65 (1.76-7.56) | **<0.001** | 0.38 (0.15-0.97) | **0.043** |
| Headache | 52, 28.6% | 40, 33.9% | 1.28 (0.78-2.11) | 0.328 | 2.17 (1.09-4.29) | 0.081 |
| Diarrhea | 31, 17% | 54, 45.8% | 4.11 (2.42-6.9) | **<0.001** | 0.51 (0.25-1.03) | 0.064 |
| Anosmia | 61, 33.5% | 91, 77.1% | 6.68 (3.94-11.34) | **<0.001** | 0.58 (0.21-1.58) | 0.292 |
| Ageusia | 61, 33.5% | 94, 79.7% | 7.77 (4.5-13.4) | **<0.001** | 0.27 (0.10-0.71) | **0.008** |
| Shortness of breath | 109, 59.9% | 89, 75.4% | 2.05 (1.23-3.43) | **0.006** | 0.53 (0.28-0.97) | **0.04** |
| Alcohol | 69, 37.9% | 41, 34.7% | 0.87 (0.54-1.41) | 0.578 | - | **-** |
| Smoker | 29, 15.9% | 38, 32.2% | 2.51 (1.44-4.36) | **0.001** | 2.50 (1.44-4.30) | 0.273 |
